# Time above range for predicting the development of type 2 diabetes

**DOI:** 10.3389/fpubh.2022.1005513

**Published:** 2022-12-08

**Authors:** Alejandra Marco, Marcos Pazos-Couselo, Jesús Moreno-Fernandez, Ana Díez-Fernández, Manuela Alonso-Sampedro, Carmen Fernández-Merino, Arturo Gonzalez-Quintela, Francisco Gude

**Affiliations:** ^1^Primary Care Center, Santiago de Compostela, Spain; ^2^Research Methods (RESMET), Health Research Institute of Santiago de Compostela (IDIS), Santiago de Compostela, Spain; ^3^Department of Psychiatry, Radiology, Public Health, Nursing and Medicine, University of Santiago de Compostela, Santiago de Compostela, Spain; ^4^Endocrinology and Nutrition Service, Ciudad Real General University Hospital, Ciudad Real, Spain; ^5^Facultad de Enfermería de Cuenca, Universidad de Castilla-La Mancha, Cuenca, Spain; ^6^Department of Clinical Epidemiology, Hospital Clínico Universitario de Santiago de Compostela, Santiago de Compostela, Spain; ^7^Primary Care Center, A Estrada, Spain; ^8^Department of Internal Medicine, Hospital Clínico Universitario de Santiago, Santiago de Compostela, Spain

**Keywords:** diabetes risk, continuous glucose monitor system, type 2 diabetes, primary care, diabetes prediction and prevention

## Abstract

**Aim:**

To investigate the prognostic value of time range metrics, as measured by continuous glucose monitoring, with respect to the development of type 2 diabetes (T2D).

**Research design and methods:**

A total of 499 persons without diabetes from the general population were followed-up for 5 years. Time range metrics were measured at the start and medical records were checked over the period study.

**Results:**

Twenty-two subjects (8.3 per 1,000 person-years) developed T2D. After adjusting for age, gender, family history of diabetes, body mass index and glycated hemoglobin concentration, multivariate analysis revealed 'time above range' (TAR, i.e., with a plasma glucose concentration of >140 mg/dL) to be significantly associated with a greater risk (OR = 1.06, CI 1.01–1.11) of developing diabetes (AUC = 0.94, Brier = 0.035).

**Conclusions:**

Time above range provides additional information to that offered by glycated hemoglobin to identify patients at a higher risk of developing type 2 diabetes in a population-based study.

## Introduction

Diabetes is characterized by a set of metabolic disorders that cause the presence of hyperglycemia in the absence of treatment. The American Diabetes Association (ADA) (1) classifies it into type 1 diabetes (T1D), type 2 diabetes (T2D), gestational diabetes and specific types of diabetes due to other causes (Latent autoimmune diabetes in adults, monogenic diabetes, among others). T2D represents 90 to 95% of all cases (1) and is characterized by hyperglycemia because of progressive resistance to the peripheral action of insulin and the possible decrease in insulin secretion associated with age (2).

The risk of developing T2D increases in people with excess weight, abdominal obesity, sedentary lifestyle, high blood pressure, low levels of high-density lipoprotein (HDL) cholesterol, hypertriglyceridemia and a lower educational level. Age, male sex and family history of diabetes are risk factors, which, unlike the previous ones, are not modifiable (3, 4). Diabetes, and specifically the degree of metabolic control, is associated with micro and macro vascular complications, which cause greater morbidity and premature mortality. In fact, people with diabetes have higher mortality rates than the population without diabetes (5, 6). Therefore, T2D represents a public health problem and a challenge for health systems and professionals involved. The early identification of patients with undiagnosed T2D and the characterization of those with a higher risk of developing a glucose metabolism disorder is essential to be able to prevent and delay the disease (5).

Continuous glucose monitoring systems (CGM) are small-sized devices that, through a subcutaneous sensor, provide information on glycemic behavior. This information allows knowing in greater detail the magnitude and duration of glycemic oscillations than with conventional measurement methods (7, 8). Although this technology is very useful for the control and monitoring of people with diabetes, CGM systems also represent a challenge for professionals, especially related to the management of the data provided and its clinical applicability. In recent years, measures related to CGM, such as glycemic variability and time in range (TIR), have been integrated into routine clinical practice (9). In 2019, a committee of experts in CGM technologies (physicians, researchers, and individuals with diabetes) published an updated consensus guide for promoting the correct and standardization use of TIR metrics in clinical practice and a more recent review about the CGM metric establishes TIR as a gold-standard measure (10, 11). In addition, the TIR has been stated as a predictor of diabetic complications (12).

CGM is clearly a very useful tool in diabetes control. However, in recent years it has also been used to study glycemic behavior in healthy volunteers (13–22). In addition to the information provided, current CGM devices are easy to use and do not interfere with activities of daily living. This makes CGM a very useful and reliable tool for research (9). The aim of this work was to investigate the possible use of glycometrics as prognostic factors for T2D development.

## Materials and methods

The A Estrada Glycation and Inflammation Study (AEGIS) is a prospective population-based study involving 1,516 subjects conducted in the municipality of A Estrada, in northwestern Spain. A summary of the project can be found at https://www.clinicaltrials.gov (code NCT01796184) and a detailed description of this population was previously published elsewhere (13).

A subsample of 622 of these subjects (41%) was randomly recruited for CGM studies. Along with the CGM, anthropometric, sociodemographic, and lifestyle data were collected. In addition, an underwent routine biochemical analysis was performed in all the involved subjects. These procedures were performed in the primary care center.

To detect those who developed T2D during the 5-year study period, FPG (fasting plasma glucose) and HbA1c (glycated hemoglobin) were determined in plasma samples. The definition of incident diabetes was based on the 2020 American Diabetes Association criteria (23). Subjects' medical records were also checked to see if any had been recorded as diabetic by their physicians over the study period.

### Study participants

#### Inclusion criteria

The study included participants with a completed CGM (at least 2 days of data) in the AEGIS study and without diabetes when the CGM was performed. For the diagnosis of diabetes, the American Diabetes Association criteria (FPG ≥ 126 mg/dL or HbA1c ≥ 6.5%) were used (13). Fasting was defined as no caloric intake for at least 8 h. In the absence of unequivocal hyperglycemia, the diagnosis of diabetes was determined with two abnormal test results in two separate test samples.

Those subjects who did not provide informed consent and/or those who were considered ineligible to participate in the study were excluded. This group included participants who were unable to perform the study procedures (dementia, intellectual disability, cerebrovascular disease, terminal cancer, or the inability to communicate). Subjects who had diabetes diagnosis at baseline and/or who presented incomplete CGM records (<2 complete days) were excluded.

### Clinical measurements. CGM procedures and data

Each participant was assigned an iPro^®^2 MiniMed^®^ CGM device equipped with an Enlite™ sensor (Medtronic, Inc, Northridge, CA, USA) inserted subcutaneously into the abdomen. The iPro^®^2 continuously measures glucose levels in the interstitial fluid and stores glucose information every 5 min, within a range of 40–400 mg/dL (2.2–22.2 mmol/L), obtaining a total of 288 glucose values per day during the useful life of the interstitial glucose sensor (6 days).

The CGM procedures (insertion, instructed the participant in the use of the CGM, removal of the sensor and data download) was performed in the primary care center by a research nurse. In addition, the participants were instructed and provided with the necessary material (glucometer, lancets, and test strips) to perform at least three measurements of capillary blood glucose per day during the CGM process. These capillary blood glucose measurements were performed to calibrate the CGM system. All participants used the same type of capillary blood glucose meter (OneTouch^®^ Verio^®^ Pro; LifeScan, Milpitas, CA, USA).

On the seventh day, the sensor was removed, and the data were downloaded and stored for further analysis. Time range metrics were gathered from the CGM data. TIR was defined as the percentage of time in the glucose range of 70–140 mg/dL (3.9–7.8 mmol/L), time below range (TBR) as the percentage of time <70 mg/dL (<3.9 mmol/L), and time above range (TAR) as the percentage of time >140 mg/dL (>7.8 mmol/L), as proposed by Zhou et al. (15), who undertook a cross-sectional study (involving CGM) on 434 individuals without diabetes.

### Clinical measurements. Metabolic disorders

At the primary care center, the participants' height, weight, and waist and hip circumference were measured. Individuals were classified as having metabolic syndrome when they met at least three of the following Adult Treatment Panel III criteria (24): (1) abdominal obesity (waist circumference > 102 cm in males or >88 cm in females); (2) hypertriglyceridaemia (fasting serum triglycerides ≥ 150 mg/dL); (3) low HDL cholesterol levels (fasting HDL cholesterol < 40 mg/dL in males or <50 mg/dL in females); (4) increased blood pressure (arterial blood pressure ≥ 130/ ≥85 mmHg or current antihypertensive medication use); and (5) hyperglycaemia (fasting serum glucose ≥ 110 mg/dL or current antidiabetic therapy. Homeostatic model assessment (HOMA-IR) was used to evaluate insulin resistance [fasting serum insulin (μU/mL) × fasting plasma glucose (mmol /L)/22.5] (25).

### Clinical measurements. Laboratory tests

At the beginning and at 5-years follow-up laboratory assessments were completed for all participants. Venous blood samples were obtained after an 8-h fasting period. HbA1c levels were determined by high-performance liquid chromatography with the use of a method certified by the National Glycohemoglobin Standardization Program. All HbA1c values were converted to Diabetes Control and Complications Trial-aligned units (26). Glucose levels were measured in the fasting serum samples using the glucose oxidase peroxidase method. Routine laboratory determinations were performed with an Olympus AU-400 analyser (Olympus, Tokyo, Japan).

### Statistical analysis

Depending on the type of distribution, the arithmetic mean or median was used as a measure of centralization, and the standard deviation or interquartile range was used as a dispersion measure.

The chi-square test to compare proportions, the Mann–Whitney test for comparison of quantitative variables and Pearson test to assess correlation were used. Multivariate analysis was used to examine the capacity of the different time range metrics to predict the development of diabetes, after adjusting for potential confounders [age, gender, family history of diabetes, BMI (body mass index) and HbA1c concentrations)]. Results are presented as odds ratio (OR) with 95% confidence intervals (CIs). Calibration was assessed using the Brier score. Model discrimination was determined by receiver operating curve analysis (AUC), and its degree of calibration *via* the Brier score.

Comparison of glucose profiles were performed using compositional and distributional functional data analysis, specifically by using glucodensities. They were estimated following the procedures by Matabuena et al. (27). For each patient, this representation estimates the proportion of time spent at each glucose concentration over a continuum.

### Ethics statement

The present study was approved by the Clinical Research Ethics Committee of Galicia, Spain (CEIC# 2012-025). Written informed consent was obtained from all subjects, in compliance with the Helsinki Declaration.

## Results

A total of 1,516 participants (44.7% males, 12.3% subjects with diabetes) were recruited into the AEGIS study. The mean age of this population was 53 ± 18 years and the mean BMI was 28.2 kg/m^2^. Regarding variables related to glucose control, mean FPG and HbA1c was 95 ± 23 mg/dL and 5.7 ± 0.8%, respectively.

**Figure 1:**
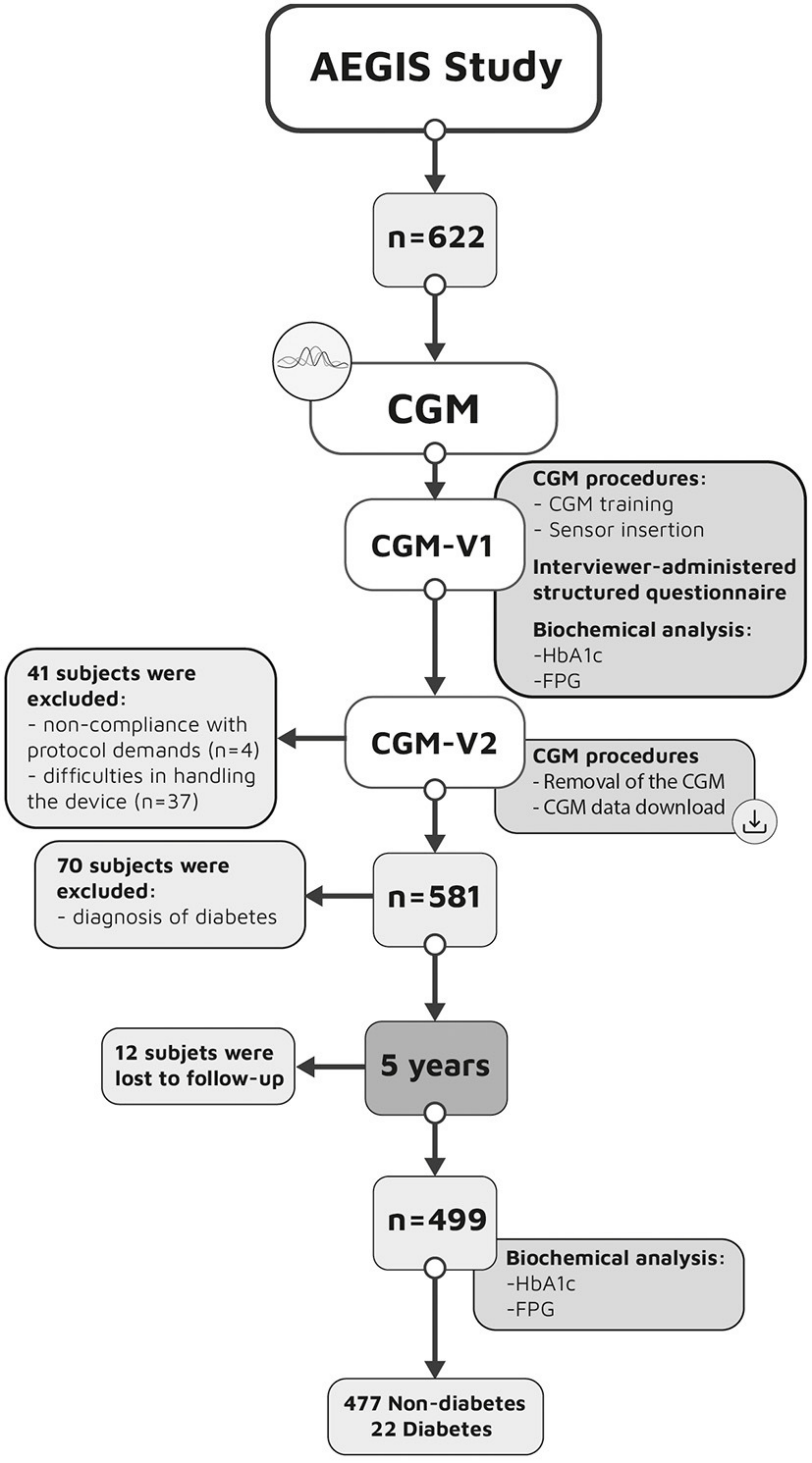
Study procedures. CGM, continuous glucose monitoring; V, visit; HbA1c, glycosylated hemoglobin; FPG, fasting plasma glucose.

For the present study, of the 622 patients assessed for eligibility, 499 qualified for participation (Figure 1). Forty one of the above 622 subjects were excluded because of non-compliance with protocol demands (*n* = 4) or difficulties in handling the device (*n* = 37). A further 70 were excluded because they already had a diagnosis of diabetes. Finally, 12 (58% males) were lost to follow-up. At baseline these subjects were younger (mean ± SD age of 37 ± 18 years), with a BMI of 26.4 ± 4.3 kg/m^2^. None of them had metabolic syndrome. Mean FPG and HbA1c was 88 ± 10 mg/dL and 5.3 ± 0.3%, respectively.

The final number of subjects with at least 2 days of monitoring (the minimum required for their data to be included in analyses) was 499. They were older (mean age 47 ± 14 years), mean BMI of 27.8 ± 5.0 kg/m^2^ and 13% had metabolic syndrome. Mean FPG and HbA1c of this subsample was 88 ± 10 mg/dL and 5.3 ± 0.3%, respectively. CGM wear time data are shown in Table 1.

**Table 1:** CGM wear time.

**CGM wear time (days)**	**Non-diabetes (*n* = 477)**	**Diabetes** **(*n* = 22)**	**LTFU subjects (*n* = 12)**
2	7 (1.5)	2 (9.1)	1 (8.3)
3	16 (3.3)	0	0
4	27 (5.6)	2 (9.1)	0
5	410 (86)	17 (77.3)	10 (83.4)
6	17 (3.6)	1 (4.5)	1 (8.3)

Data are expressed as absolute frequencies and percentages. CGM, continuous glucose monitoring system; LTFU, lost to follow-up.

Of the 499 subjects whose data were eligible for analysis, 22 (7 males) developed diabetes over the 5-year follow-up period a cumulative incidence of 4.4%. The raw incidence rate in the sample was 8.3 per 1,000 person-years (95%CI 5.2–12.6).

Univariate analysis showed that the subjects who developed diabetes were older and showed initially higher values for BMI, serum FPG and HbA1c, than did those who remained non-diabetic. Metabolic abnormalities were also more common among the subjects who developed diabetes (Table 2).

**Table 2:** Baseline clinical characteristics of subjects according to the development of type 2 diabetes after 5-years follow-up.

		**Non-diabetes (*n* = 477)**	**Diabetes** **(*n* = 22)**	***P*** **-value**
Age, years (SD)		46.4 (13.8)	54.8 (10.0)	<0.001
Males (%)		168 (35)	7 (32)	0.747
Body mass index, Kg/m2 (SD)		27.6 (4.9)	33.2 (4.8)	<0.001
Family history of diabetes (%)		241 (50)	14 (64)	<0.001
Fasting plasma glucose, mg/dL (SD)		87 (10)	105 (12)	<0.001
HbA1c, % (SD)		5.3 (0.3)	5.8 (0.7)	<0.001
mmol/mol (SD)		34.8 (3.4)	39.9 (4.2)	
HOMA-IR, μUI/mL × mmol/L (SD)		2.55 (1.69)	5.32 (3.31)	<0.001
	Abdominal obesity (%)	171 (36)	19 (86)	<0.001
	Hyperglycaemia^*^ (%)	18 (4)	9 (41)	<0.001
**Metabolic syndrome** ^‡^	Hypertriglyceridaemia (%)	78 (16)	6 (27)	0.108
	Low HDL cholesterol levels (%)	79 (17)	6 (27)	0.194
	High blood pressure (%)	174 (36)	17 (77)	<0.001
	Metabolic syndrome (%)	54 (11)	13 (59)	<0.001

Data are absolute frequencies and percentages, or means and standard deviations (within parentheses). SD, standard deviation; HOMA-IR, Homeostatic Model Assessment of Insulin Resistance; TBR, time below range; TIR, time in range; TAR, time above range.

‡ Criteria from the Adult Treatment Panel III for the definition of metabolic syndrome: abdominal obesity, defined by waist circumference > 102 cm in males or >88 cm in females; hypertriglyceridaemia, defined by fasting serum triglycerides ≥ 150 mg/dL; low high-density lipoprotein (HDL)-cholesterol levels, defined by fasting HDL-cholesterol < 40 mg/dl in males or <50 mg/dl in females; high blood pressure, defined by blood pressure ≥ 130/ ≥85 mmHg or current anti-hypertensive medication use; and hyperglycaemia, defined by fasting blood glucose ≥ 110 mg/dL.

* Subjects diagnosed of diabetes are excluded. Individuals meeting at least three of these criteria were considered to have metabolic syndrome.

Over the monitoring period, TBR was close to 0% in most subjects. The TAR value was significantly higher in those who developed diabetes [median 7.8% (interquartile range, IQR 4.5–20)] than in those who remained non-diabetic [median 1.9% (IQR 0.4–5.1), *p* < 0.001]. The TIR value for those who remained non-diabetic was significantly greater than for those who developed diabetes (*p* < 0.001) (Table 3).

**Table 3:** Baseline continuous glucose monitoring data of subjects according to the development of type 2 diabetes after 5-years follow-up.

	**Non-diabetes (*n* = 477)**	**Diabetes** **(*n* = 22)**	***P*** **-value**
TBR (%)	0 [0, 1.7]	0 [0, 1.2]	0.268
TIR (%)	96.4 [92.6, 99.1]	91.1 [77.5, 94.3]	<0.001
TAR (%)	1.9 [0.4, 5.1]	7.8 [4.5, 20.0]	<0.001

Data are absolute frequencies and percentages, or means and standard deviations (within parentheses), or medians [Percentil25, Percentil75]. TBR, time below range; TIR, time in range; TAR, time above range. TBR is defined as a percentage of values < 70 mg/dL (< 3.9 mmol/L). TIR is defined as a percentage of values between 70 and 140 mg/dL (3.9–7.8 mmol/L), %. TAR is defined as a percentage of values >140 mg/dL (>7.8 mmol/L).

**Figure 2:**
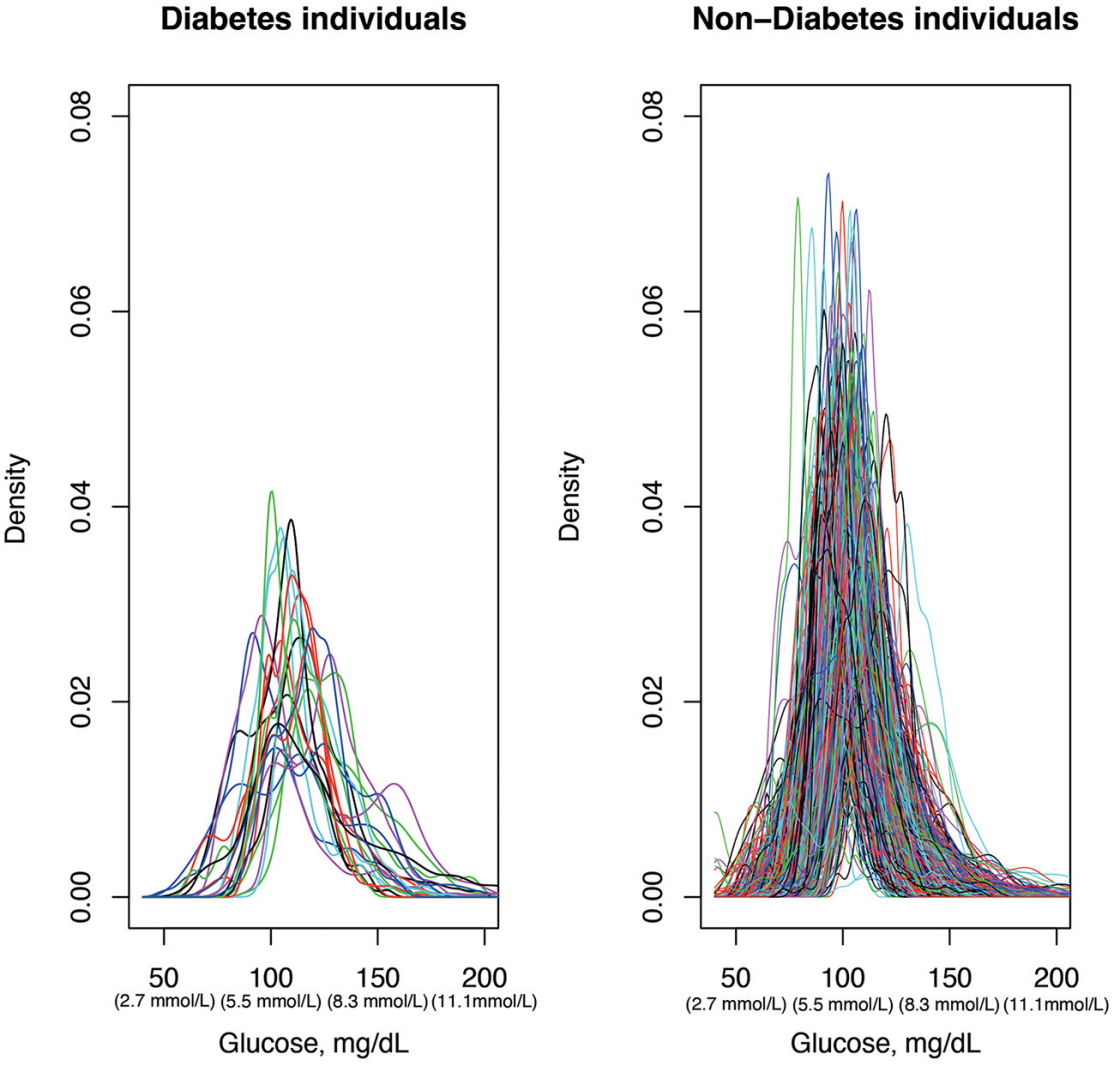
Glucodensities. Interstitial glucose data obtained from continuous glucose monitoring. For each patient, glucose representation estimates the proportion of time spent at each glucose concentration over a continuum.

**Figure 3:**
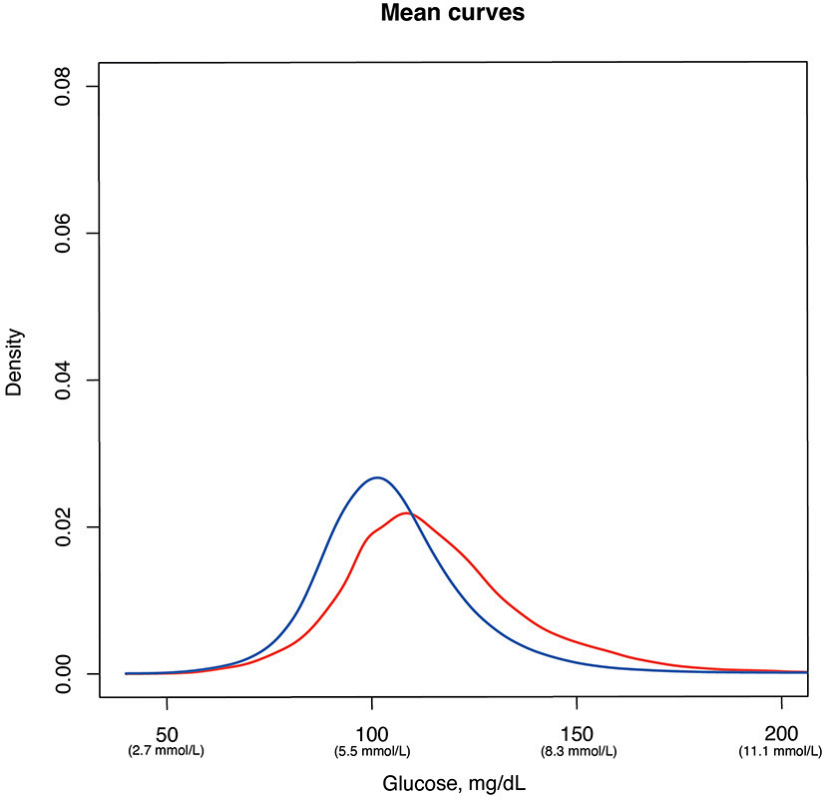
Univariate density distributions of continuous glucose monitoring data in subjects with diabetes (red line) and without diabetes (blue line).

Glucodensities (Figure 2) showed that, those subjects who developed T2D after the 5-years follow-up period presented at baseline a lower density in values around 100 mg/dL (5.6 mmol/L) and a slight shift to the left of glucose curves. The same effect was observed in the analysis of the mean curve of the densities (Figure 3), in which a statistically significant difference was found (*p* = 0.034) between subjects who developed diabetes after the 5-years follow-up period and those who did not.

After adjusting for age (*p* = 0.199), gender (*p* = 0.256), a family history of diabetes (*p* = 0.025), BMI (*p* = 0.003) and HbA1c concentration (*p* < 0.001), multivariate analysis confirmed TAR to be a good prognostic marker of the development of diabetes (OR = 1.06, 95% CI 1.01–1.11; *p* = 0.033). The AUC for the regression model was 0.94 (95% CI 0.89, 0.98); its calibration was stable (Brier score = 0.035). TAR values correlated strongly with FPG (Pearson R = 0.76; *P* < 0.001), and HbA1c (R = 0.83; *P* < 0.001).

## Discussion

This study is the first that has examined the risk of developing diabetes using time range information obtained from CGM data. Our results indicate that TAR can identify persons in the general population who are at risk of developing T2D within 5-years, even after adjusting for age, sex, BMI, family history of diabetes and HbA1c. The incidence of diabetes was 8.3 per 1,000 person-years, similar to that found in other prospective, population-based studies (28, 29).

The prevention of T2D is a challenge for health systems around the world. Therefore, those tools that allow health professionals to identify risk factors for developing diabetes should be included in routine clinical practice. In addition, identifying people at higher risk is essential to develop interventions on lifestyles efficiently (5).

Although there are many risk prediction models for type 2 diabetes, only a small minority are used in routine clinical practice. Furthermore, in a systematic review on risk prediction models for T2D (5), the authors highlight the widespread use of poor methods that could affect the reliability and thus the clinical use of prediction models. The most used prediction variables in studies and in clinical practice are age, family history of diabetes, BMI, hypertension, waist circumference and sex. Other commonly identified risk predictors included ethnicity, fasting glucose level, smoking status, and physical activity (5). Therefore, it seems necessary to develop new assessment tools to include them in clinical practice and that provide reliable information on the risk of developing diabetes.

CGM systems are a technology through which we can obtain data on glucose profiles in a complex and reliable way. This information allows the magnitude and duration of glycemic oscillations to be measured more precisely than conventional measurement systems and its integration into routine clinical practice has increased considerably in recent years (30). There are numerous studies (clinical trials and real-world observational studies) that show the clinical benefits of continuous glucose monitoring in patients with T1D and T2D (mainly treated with insulin) (31–44). However, there is a growing interest in expanding the use of CGM regardless of type of diabetes or treatment regimen (45). In addition, the technical characteristics of CGM devices make this technology very useful for studying glycemic behavior in populations without diabetes (13–21), and a better indicator of possible early dysglycaemia than either FPG or HbA1c (22).

The most recent technological developments and the main clinical use of CGM systems have focused on real-time information (glucose values and trends and prediction of hypo and hyperglycemia). However, it is important to note that CGM also provides retrospective information on glycemic behavior. The clinical utility of retrospective data analysis is to identify off-target glucose patterns, determine potential causes, and discuss possible solutions (46). In addition to clinical use, retrospective analysis is very useful in research.

Measuring the impact of the time spent at each glucose concentration and the associated glucose oscillations is crucial for the assessment of glucose metabolism with a high degree of precision (27). Although CGM systems allow us to obtain the data to understand and analyze glucose profiles, it is also important to integrate this type of measurement into clinical practice. Therefore, recent studies have shown the need for using other types of glycemic measurements that we can obtain with the CGM data, such as TIR. TIR is strongly associated with the risk of microvascular complications and should be an acceptable endpoint for clinical trials (12, 14, 47).

The present findings support the routine clinical use of CGM and suggest TAR could be valuable in helping to identify persons at risk of developing diabetes independent of their HbA1c concentration (48). Unlike the HbA1c, TAR is better capturing acute glucose spikes occurring after meals. We previously showed that after any given meal (breakfast, lunch or dinner) glucose values > 140 mg/gL (7.8 mmol/L) were attained by 7% subjects with normal glycemia and 20% with prediabetes (48).

Although more studies are needed to define the characteristics of the subjects who would benefit from performing a CGM to assess the risk of developing diabetes the reliability, ease of use and the information provided by CGM systems turns these devices into a complementary tool that could help improve prediction models and that could be included in clinical practice in the context of primary care. However, the use of CGM in this context should not be considered for a population screening in which a general implementation is carried out. Therefore, it is necessary to define which population could benefit from this technology when measuring the risk of developing diabetes. In view of the results of our work, it seems that people over 50-years of age, with metabolic syndrome (especially abdominal obesity), high blood pressure and who have FPG values >100 mg/dL and/or HbA1c values > 5.7% would be candidates for perform a CGM to assess the risk of diabetes and implement prevention strategies that include a better selection of patients and individualized and more efficient clinical recommendations (for example, focus on the periods of the day in which there is a higher TAR). Other authors have also assessed the usefulness of CGM in primary care, although it is mainly focused on the follow-up and control of patients with diabetes (30, 45).

Regarding the monitoring time, although the recommendations to optimize clinical decisions in diabetes are 14 days (9) a recent study (49) showed that the correlation between the 14 and 7-day glucose management indicator (GMI) was 0.95 and the correlation between 14 vs. 10, 5, and 3 days were 0.98, 0.91, and 0.86, respectively. In addition, these recommendations are focused on the use of CGM to make clinical decisions and follow-up of patients with diabetes. In our work, although in the inclusion criteria we had set a minimum of 2 complete days of CGM data, of the 499 subjects analyzed, the majority (89.2%) completed at least 5 days. Considering the results found it seems reasonable to indicate a similar monitoring time, however current CGM systems allow data to be obtained between 10 and 14 days without the need to change the device.

Furthermore, while the proposed TAR cut-off for identifying those at risk of developing diabetes is certainly useful, predictions might be strengthened if continuous glucose density values could be used instead, as recently described (27). Thus, in addition to the TIR/TAR/TBR analysis, we have assessed the differences in the CGM profiles by means of density. The glucodensities are a new functional representation of CGM with a high association between HbA1c, HOMA-IR and glycemic variability parameters (Continuous Overlapping Net Glycemic Action, Mean of Daily Differences, Mean Amplitude of Glycemic Excursions) (27). This type of measurement of glycemic behavior through CGM data allows to have a simple and more accurate representation of the glycemic profile of an individual. This representation is especially useful in to establish if there are statistically significant differences between patients based on their glycemic condition and other variables, as well as to analyze the relationship of an individual's glycemic profile with different clinical variables in epidemiological studies (27).

In our work we have observed differences between the subjects who developed diabetes after 5-years and the group that did not change their glycemic status when we analyze the density of the glucose profiles. Although more studies are needed to assess the reliability and usefulness of glucodensities in the population with and without diabetes, the use of this type of measurement tools and their inclusion in conventional glucometers would contribute to a more complex assessment of glucose profiles.

### Strengths and limitations

The main strength is that our investigation is based on the use of CGM as a tool of clinical use for the prediction of the development of T2D. Our study highlights in the proposal of a new method for assessing the risk of diabetes based on the values of the TIR and a representation of glucose profiles using distributional data analysis (glucodensities).

This study is limited in that the sample size and the follow-up time were relatively short for investigating the risk of developing diabetes. Finally, while the study was conducted in the general population, it involved only data from the population of A Estrada; further work will be needed to check the representativeness of the present findings in other areas.

### Conclusions

CGM systems are a tool that better allow assessing and knowing the glycemic behavior. For a complete evaluation of the glucose profiles, additionally to the time in range, in CGM data it should be included parameters that go beyond the average glucose values and that also value accurately glycemic variability.

Due to the characteristics of CGM systems and their clinical applicability, the use of these devices should be generalized in primary care.

Based on our results, we can conclude that time above range and glucodensities, as measured by CGM, could provide prognostic markers for identifying those at a higher risk of developing T2D.

## Data availability statement

The original contributions presented in the study are included in the article/supplementary material, further inquiries can be directed to the corresponding author.

## Ethics statement

The studies involving human participants were reviewed and approved by Clinical Research Ethics Committee of Galicia, Spain (CEIC 2012/025 and CEIC 2016/240). The patients/participants provided their written informed consent to participate in this study.

## Author contributions

Conception of the study: FG and AG-Q. Study design: MP-C, JM-F, AD-F, MA-S, FG, and AG-Q. Data collection: AM, CF-M, and MP-C. Data analysis: FG. All authors helped to check the various drafts of the manuscript, editing, and approved the manuscript.
